# Analysis of the spectrum of critical illnesses in the emergency department of Hebei Children's Hospital: A single-center study

**DOI:** 10.1371/journal.pone.0360675

**Published:** 2026-10-09

**Authors:** Xiao Wang, Weili Guo, Yishan Guo, Pengli Hao, Pei Zhao, Gailing Hao

**Affiliations:** 1 Emergency Department, Hebei Children's Hospital, Hebei Clinical Research Center for Children's Health and Diseases, Shijiazhuang City, Hebei province, China; 2 Hebei North College, Zhangjiakou City, Hebei province, China; Fayetteville State University, UNITED STATES OF AMERICA

## Abstract

**Objective:**

To analyze the distribution and epidemiological characteristics of critical illnesses in the emergency department of Hebei Children's Hospital.

**Method:**

Through retrospective medical record review, patients triaged as level Ⅰ or Ⅱ (aged 0–18 years) who visited the emergency department of the hospital from January 2023 to December 2024 were included. The distribution of diseases, age, gender, season and other indicators were statistically analyzed.

**Results:**

A total of 1,450 critically ill patients with triage levels Ⅰ and Ⅱ were included. The median (IQR) age of all patients was 4.00 (1.00, 9.00) years, and the male-to-female ratio was 1.2:1. Among each age group, the 1 to <3 years group had a proportion of 27.9%, and the 0 to <1 year group had a proportion of 9.5%, representing the highest and lowest values, respectively. The distribution of critical illnesses in the emergency department of this hospital was led by the nervous system (n = 548, 37.8%), trauma and accidental injuries (n = 286, 19.7%), and the respiratory system (n = 214, 14.8%), which ranked as the top three categories. The top five primary diagnosed diseases were seizure (n = 352, 24.3%), drug poisoning (n = 242, 16.7%), severe pneumonia (n = 137, 9.5%), status epileptics (n = 137, 9.5%), and arrhythmia (n = 102, 7.0%). Among them, the proportion of severe pneumonia was the highest in the 0 to <1 year group; the proportion of seizure was the highest in the 1 to <3 years group, 3 to <6 years group and 6 to <12 years group, while the proportion of drug poisoning was the highest in the 12 to <18 years group. The distribution of the top five disease differed significantly across age groups (*p* < 0.001) and seasons (*p* = 0.004). Cardiopulmonary arrest (CPA) is one of the most critical conditions in an emergency department. However, the distribution of diseases causing CPA did not show significant differences by age or season. The top three diseases causing CPA are severe pneumonia, asphyxia, and multiple body region injuries (Road traffic accident)**.**

**Conclusion:**

The spectrum of critical illnesses varied significantly by age and seasonal distribution in the emergency department of Hebei Children's Hospital. Therefore, age and season-tailored approaches are recommended for managing common critical illnesses, whereas CPA requires a standardized, universally applied emergency response. It is necessary to further strengthen training and optimize resource allocation.

## Introduction

Pediatric critical illness, such as sepsis, severe pneumonia, and status epileptics, constitutes a major clinical and public health burden worldwide, and accounts for a high proportion of child deaths [1–5]. Pediatric emergency department is the forefront of the treatment of critically ill children, which requires rapid identification of critical illness and rapid decision-making. Its diagnosis and treatment ability is closely related to the survival rate and long-term prognosis of children [6,7]. Respiratory tract infection, sepsis, and trauma have long been recognized as the main causes of pediatric emergency department visits and critical illness [8,9]. However, with the development of social economy and environmental changes, the disease spectrum of pediatric emergency department is also changing [10–12]. At the same time, comprehensive and up-to-date data on the disease spectrum of pediatric emergency departments in Hebei Province, a populous province in northern China, are still obviously insufficient [13]. Hebei Children's Hospital, as the largest tertiary medical center in the province, with an annual outpatient and emergency volume of about 1.7 million, provides a valuable opportunity to fill this knowledge gap. Therefore, this study aimed to systematically describe the spectrum of critical illness presenting to the emergency department of Hebei Children's Hospital, analyze its age and seasonal distribution, and analyze the causes of cardiopulmonary arrest (CPA) in children, so as to provide region-specific evidence for clinical practice, emergency preparedness, and health policies in similar settings.

## Materials and methods

### Study design and setting

This study used a retrospective, observational, single-center design, and the study site was the emergency department of Hebei Children's Hospital. As the largest pediatric referral center in the province, the hospital serves more than 10 million people, covering both urban and rural areas. The hospital has 1,800 beds, and has 30 beds in pediatric intensive care unit (PICU), neonatal intensive care unit (NICU) and other critical care resources [14]. Children and adolescents aged from birth to 18 years were included in the study. The annual volume of emergency department visits is about 110,000–140,000 [15]. In order to identify critical cases, the emergency department divided the patients into critical, severe, urgent and non-urgent groups according to the standard of “Emergency triage Classification Management” formulated by the expert consensus on pediatric emergency pre-examination [16] and triage combined with the actual situation of the hospital, corresponding to level Ⅰ, level Ⅱ, level Ⅲ and level Ⅳ respectively. Among them, level Ⅰ and level Ⅱ were the subjects of this study. The medical team is composed of qualified pediatric emergency physicians and professionally trained nurses, and implements a 24-hour on-duty system. In terms of geographical and environmental characteristics, Hebei province has a temperate continental monsoon climate. The region has cold and dry winters (mean temperature −5°C to 0°C) and hot and humid summers (mean temperature 25°C to 30°C), and significant seasonal climate fluctuations have important effects on the epidemiological patterns of respiratory and infectious diseases [17,18]. The service population is highly diverse, covering urban and rural residents with different socioeconomic status and medical accessibility, which can reflect the whole picture of regional medical challenges [19]. Data were extracted from the hospital electronic medical record (EMR) system and the pediatric emergency registry. This system completely recorded all the information of children in the emergency department, including demographic characteristics, clinical diagnosis, treatment procedures and final outcomes, which provided a reliable basis for data analysis.

### Study population and sampling

We identified all consecutive patients aged 0−18 years who visited the emergency department between January 1, 2023, and December 31, 2024, through a review of hospital electronic medical record systems. Among the 249,136 emergency visits during this period, patients with a triage level I or II were initially selected as potential participants. Inclusion criteria: (1) age 0−18 years; (2) Triage level I or II; (3) complete medical records (including diagnosis, treatment, and outcome data). The exclusion criterion was incomplete medical records (lacking any of the above key data). After applying these criteria, 1,517 critically ill patients were identified. Of these, 67 were excluded due to incomplete records, and 1,450 critically ill patients were included in the final analysis. This study was approved by the Ethics Committee of Hebei Children's Hospital (Approval Number: 202222−29).

### Data indicators and standards

In this study, critical illness was defined as conditions or symptoms with a triage level of I or II. Level I indicated life-threatening conditions requiring immediate intervention (e.g., CPA, status epilepticus); Level II referred to high-risk but not immediately life-threatening conditions requiring urgent intervention (e.g., acute poisoning, respiratory distress). The term “disease” was used as a general term, encompassing not only specific pathological conditions (e.g., pneumonia, epilepsy) but also traumatic injuries (e.g., fractures, traumatic intracranial hemorrhage), symptoms (e.g., convulsions, coma), and external causes of morbidity (e.g., road traffic accidents, falls from height), all of which were derived from the triage records in the emergency department. This broad application reflects the heterogeneity of emergency department visits and is consistent with the coding framework of the International Classification of Diseases (ICD). Cardiopulmonary arrest (CPA) was defined as the absence of spontaneous breathing and a palpable pulse, requiring chest compressions, endotracheal intubation, and/or defibrillation. CPA cases were identified through ICD-10 code I46.901 and manual review of resuscitation records. Two reviewers independently confirmed each CPA event. Return of spontaneous circulation (ROSC) was defined, according to published criteria, as the return of a palpable pulse and measurable blood pressure sustained for ≥20 minutes after CPA [20,21]. Patients who achieved ROSC were assigned to the ROSC group; those who did not were assigned to the death group.

Variables collected:

Demographics: Age [categorized into five groups: 0 to <1 year (infancy), 1 to <3 years (early childhood), 3 to <6 years (preschool age), 6 to <12 years (school age), and 12 to <18 years (adolescence)] and gender.

Triage level: Level I, II, III, and IV.

Disease classification: All diagnoses were coded using the International Classification of Diseases, 10th Revision (ICD-10).

Season: Defined as spring (March to May), summer (June to August), autumn (September to November), and winter (December to February).

All data were retrospectively extracted from the emergency department electronic medical record (EMR) system. To ensure data accuracy and completeness, two trained research assistants independently reviewed each record, and any discrepancies were resolved by consensus with a third senior researcher. Patients with incomplete key variables (e.g., missing diagnosis) were excluded from the analysis (n = 67).

### Statistical analysis

Excel 2021 (Microsoft Corporation, Redmond, WA, USA) [22] was used for preliminary data management; GraphPad Prism 9 (GraphPad Software, San Diego, CA, USA) [23] was used for graphing, and SPSS 26.0 (IBM Corp., Armonk, NY, USA) [24] was used for statistical analysis. The distribution of continuous variables was assessed by visual inspection of histograms and Q-Q plots and by skewness and kurtosis values; given the large sample size (n > 1450), formal normality tests were not relied upon because they are oversensitive to minor deviations. Variables with substantial skew were summarized as median (IQR) and analyzed with non-parametric methods. Comparisons of continuous variables between groups were performed using the Mann-Whitney U test (for two groups) or the Kruskal-Wallis H test (for more than two groups). Categorical data were presented as frequencies and percentages. Comparisons of categorical variables were performed using the chi-square test or Fisher's exact test. For statistically significant chi-square tests, adjusted standardized residuals were calculated, and residuals with an absolute value≥1.96 were considered the main source of differences. Bonferroni correction was applied to control the family-wise error rate for multiple comparisons. A two-sided *p* < 0.05 was considered statistically significant.

## Study results

### Baseline characteristics of critical illnesses

Among the 1450 critically ill patients, there were 804 male cases (55.4%) and 646 female cases (44.6%), with a male-to-female ratio of 1.2:1. The median age was 4.00 (1.00, 9.00) years. Among all age groups, the 1 to <3 years group had the highest constituent ratio (404, 27.9%), followed by the 6 to <12 years group (385, 26.6%), and the 0 to <1 year group had the lowest constituent ratio (138, 9.5%). There was a significant difference in the proportion of males and females in each age group (χ² = 19.150, *p* = 0.001). As shown in Table 1.

**Table 1:** Gender and age distribution of critical illnesses, n (%).

Gender	Age groups					Total
	0 to <1 yr	1 to <3yr	3 to <6yr	6 to <12yr	12 to <18yr	
Male	84(10.4)	216(26.9)	204(25.4)	213(26.5)	87(10.8)	804(55.4*)
Female	54(8.4)	188(29.1)	122(18.9)	172(26.6)	110(17.0)	646(44.6*)
Total	138(9.5)	404(27.9)	326(22.5)	385(26.6)	197(13.6)	1450(100)
χ²	19.150					
*p*	0.001					

Note:* The percentages of males and females in the total population, respectively; χ² test for association between gender and age-group distribution (df = 4); yr: year/years.

### Distribution of disease systems in different age groups

As shown in Table 2, the proportion of nervous system diseases was the highest (548, 37.8%), followed by trauma and accidental injury (286, 19.7%), and the third was respiratory system diseases (214, 14.8%). The highest proportion in the 0 to <1 year group was respiratory diseases (60, 43.5%). In the 1 to <3 years group, 3 to <6 years group, and 6 to <12 years group, the highest proportion in all three groups was nervous system diseases (43.6%, 51.5%, and 35.3%, respectively). The 12 to <18 years group had the highest proportion of trauma and accidental injury (53, 26.9%). There were 58 cases of multi-system diseases, accounting for 4.0%.

**Table 2:** Disease system distribution according to age group, n (%).

System distribution	Age groups					Total
	0 to <1 yr	1 to <3yr	3 to <6yr	6 to <12yr	12 to <18yr	
Nervous System	26(18.9)	176(43.6)	168(51.5)	136(35.3)	42(21.3)	548(37.8*)
Trauma and Injury	5(3.6)	134(33.2)	58(17.8)	36(9.4)	53(26.9)	286(19.7*)
Respiratory System	60(43.5)	55(13.6)	34(10.4)	51(13.2)	14(7.1)	214(14.8*)
Circulatory System	13(9.4)	22(5.5)	25(7.7)	58(15.1)	31(15.8)	149(10.3*)
Endocrine Metabolism	5(3.6)	5(1.2)	19(5.8)	53(13.8)	31(15.8)	113(7.8*)
Digestive System	5(3.6)	5(1.2)	5(1.5)	14(3.6)	5(2.5)	34(2.3*)
Immune System	5(3.6)	0(0.0)	6(1.9)	13(3.4)	4(2.0)	28(1.9*)
Hematologic System	1(0.7)	1(0.2)	4(1.2)	5(1.3)	4(2.0)	15(1.0*)
Urinary System	1(0.7)	0(0.0)	0(0.0)	2(0.5)	2(1.0)	5(0.3*)
Multiple System	17(12.3)	6(1.5)	7(2.2)	17(4.4)	11(5.6)	58(4.0*)
Total	138(100)	404(100)	326(100)	385(100)	197(100)	1450(100)

Note:* The percentage of cases of each disease system relative to the total study population; yr: year/years.

### Overall distribution of the disease spectrum

As shown in Table 3, the main diagnostic spectrum included 20 disease types with more than 10 cases, among which seizures accounted for the largest proportion (352, 24.27%), followed by drug poisoning (242, 16.69%) and severe pneumonia (137, 9.45%). There were 25 disease types with fewer than 10cases, mainly heart failure, intussusception, intestinal perforation, etc. From Fig 1, we can see that there are gender differences in the disease spectrum. The incidence of anaphylaxis, sepsis, laryngeal obstruction, multiple body region injuries(road traffic accident), electrolyte disturbance, anemia and gastrointestinal bleeding in boys was significantly higher than that in girls. The incidence of intracranial hemorrhage in boys was significantly lower than that in girls.

**Table 3:** Overall distribution of the spectrum of critical diseases.

Encoding	Disease spectrum (ICD-10 code)	2023 n(%)	2024 n(%)	Total n(%)
Ⅰ	Seizure (R56.803)	176(12.1)	176(12.1)	352(24.3)
Ⅱ	Drug poisoning (T50.900x001)	91(6.3)	151(10.4)	242(16.7)
Ⅲ	Severe pneumonia (J18.903)	70(4.8)	67(4.6)	137(9.5)
Ⅳ	Status epilepticus (G41.900)	61(4.2)	76(5.2)	137(9.5)
Ⅴ	Arrhythmia (I49.900)	44(3.0)	58(4.0)	102(7.0)
Ⅵ	Diabetic ketoacidosis(E14.100x011)	31(2.1)	49(3.4)	80(5.5)
Ⅶ	Coma (R40.200)	26(1.8)	18(1.2)	44(3.0)
Ⅷ	Respiratory failure (J96.900)	17(1.2)	19(1.3)	36(2.5)
Ⅸ	Cardiopulmonary Arrest (I46.901)	18(1.2)	17(1.2)	35(2.3)
Ⅹ	Shock (R57.900)	18(1.2)	15(1.0)	33(2.3)
XI	Anaphylaxis (T78.400x002)	13(0.9)	15(1.0)	28(2.0)
XII	Hypoglycemia (E16.200)	10(0.7)	11(0.8)	21(1.5)
ⅩⅢ	Intracranial infection (G06.006)	7(0.5)	12(0.8)	19(1.3)
ⅩⅣ	Laryngopharyngitis (J38.601)	3(0.2)	13(0.9)	16(1.1)
ⅩⅤ	Sepsis (A41.901)	10(0.7)	6(0.4)	16(1.1)
ⅩⅥ	Multiple body region injuries(Road traffic accident)(T06.800x001)	6(0.4)	10(0.7)	16(1.1)
ⅩⅦ	Traumatic intracranial hemorrhage (S06.804)	7(0.5)	8(0.5)	15(1.0)
ⅩⅧ	Electrolyte disturbance (E87.800x011)	7(0.5)	6(0.4)	13(0.9)
ⅩⅨ	Anemia (D64.900)	7(0.5)	6(0.4)	13(0.9)
ⅩⅩ	Gastrointestinal hemorrhage (K92.210)	4(0.3)	8(0.5)	12(0.8)
XXI	Others*	48(3.3)	35(2.4)	83(5.7)
	**Total**	674(46.5)	776(53.5)	1450(100)

**Note:** * Indicates the spectrum of diseases with fewer than 10 episodes, including heart failure, intussusception, bowel perforation, crush injury, falling, jumping, or being pushed (with unclear intent) from a height, renal insufficiency, drowning, pulmonary hypertension, death, etc.

**Fig 1:**
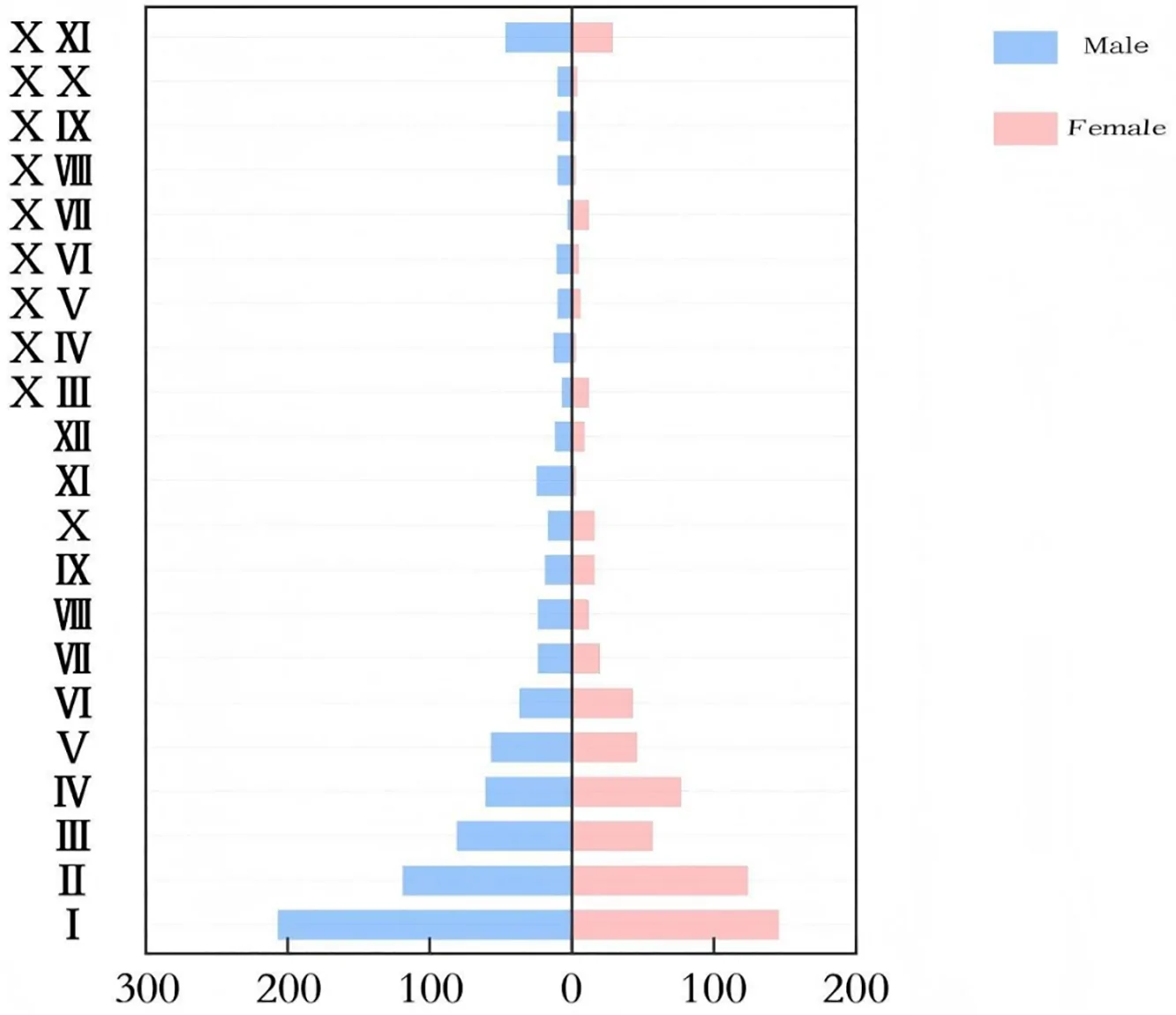
Disease spectrum in the Critically illness patients.

### Disease spectrum-age association analysis

As shown in Table 3, the top five diseases in the spectrum of critical illnesses were seizure, drug poisoning, severe pneumonia, status epilepticus, and arrhythmia. Fig 2 presents the distribution of the top five disease profiles for each age group. The 0 to <1 year group had the highest proportion of severe pneumonia. The 1 to <3 years, 3 to <6 years and 6 to <12 years groups had the highest proportion of seizures, while the 12 to <18 years group had the highest proportion of drug poisoning. As shown in Table 4, there were significant differences in the distribution of the top five diseases among different age groups (χ^2^ = 297.767, *p <* 0.001). Post hoc analysis (based on adjusted residuals) showed that the incidence of seizures was significantly higher in the 1 to <3 years group than in the other groups (*p* < 0.01), and the incidence of seizures was lowest in the 12 to <18 years group (*p* < 0.01). The incidence of drug poisoning in the 1 to <3 years and 12 to <18 years groups was significantly higher than that in the other three groups (*p* < 0.01), while the incidence in the 0 to <1 year and 6 to <12 years groups was significantly lower. The incidence of severe pneumonia in the 0 to <1 year group was significantly higher than that in other age groups (*p* < 0.01), while the incidence of severe pneumonia in 3 to <6 years group was significantly lower (*p* < 0.01). The proportion of status epilepticus in the 3 to <6 years group and 6 to <12 years group was significantly higher than that in the other three groups (*p* < 0.01), and the incidence of status epilepticus in the 1 to <3 years group was significantly lower (*p* < 0.01). The proportion of arrhythmia in the 6 to <12 years group was significantly higher than that in the other age groups (*p* < 0.01), and the incidence of arrhythmia in the 1 to <3 years group was significantly lower (*p* < 0.01).

**Table 4:** Differences in the distribution of the top five diseases profiles by age group.

Diseases	Index	Age groups					Total
		0 to<1 yr	1 to <3yr	3 to <6yr	6 to <12yr	12 to <18yr	
Seizure	n(%)	17(4.8)	154(43.8)	104(29.5)	61(17.3)	16(4.5)	352(100)
	Adjusted residuals	−2.2*	3.7**	2.3*	−1.9	−4.7**	
Drug poisoning	n(%)	1(0.4)	124(51.2)	50(20.7)	19(7.9)	48(19.8)	242(100)
	Adjusted residuals	−4.8**	5.6**	−1.9	−5.6**	5.3**	
Severe pneumonia	n(%)	42(30.7)	37(27.0)	20(14.6)	31(22.6)	7(5.1)	137(100)
	Adjusted residuals	11.3**	−2.4*	−3.1**	0.7	−2.3*	
Status epilepticus	n(%)	6(4.4)	21(15.3)	53(38.7)	44(32.1)	13(9.5)	137(100)
	Adjusted residuals	−1.4	−5.5**	3.9**	3.6**	−0.5	
Arrhythmia	n(%)	5(4.9)	15(14.7)	18(17.6)	44(43.1)	20(19.6)	102(100)
	Adjusted residuals	−1.0	−4.8**	−1.9	6.0**	3.1**	
Statistic	n=970, χ²=297.767, df=16, *p***﹤**0.001, Cramer's V=0.277						

**Note:** * Indicates the absolute value of the adjusted residual value ≥ 1.96 (*p* < 0.05); ** Indicates the absolute value of the adjusted residual value ≥ 2.58 *(p* < 0.01); yr: year/years.

**Fig 2:**
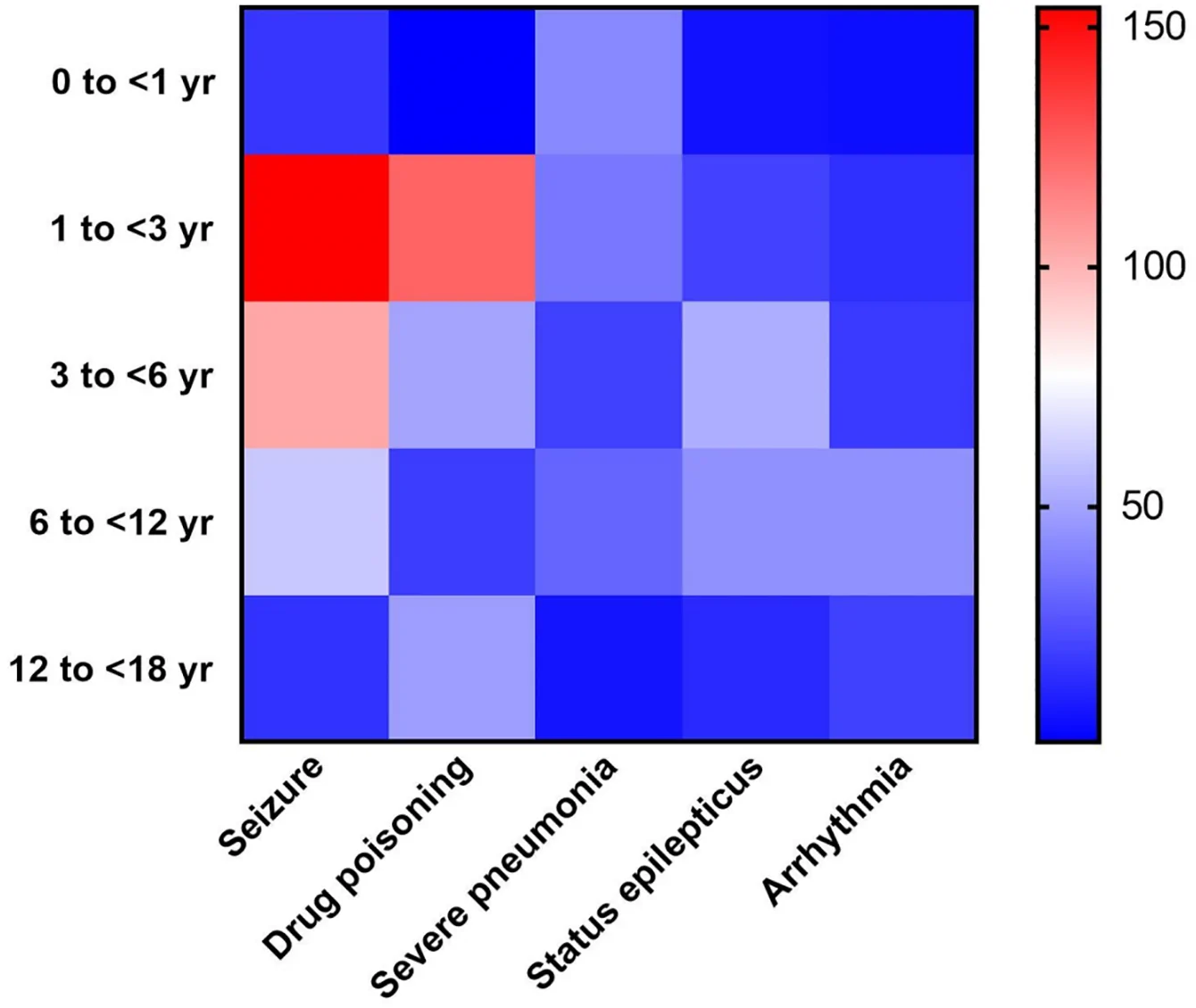
Distribution of the top five disease profiles according to age group.

### Disease spectrum-seasonal association analysis

Table 5 shows that the seasonal distribution of the top five disease spectra was significantly different (χ^2^ = 28.877, *p* = 0.004). The incidence of drug poisoning was the highest in spring (*p* < 0.001). The incidence of severe pneumonia in summer was higher than that in other seasons *(p* < 0.05), and the incidence was the lowest in spring *(p* < 0.05). The incidence of arrhythmia was highest in autumn (*p* < 0.01). However, the seasonal distribution of seizures and status epilepticus was not significant.

**Table 5:** Differences in the distribution of the top five diseases in different seasons.

Disease	Index	Seasons				Total
		Spring	Summer	Autumn	Winter	
Seizure	n(%)	66(18.8)	102(29.0)	88(25.0)	96(27.3)	352(100)
	Adjusted residuals	−1.6	−0.1	−0.1	1.8	
Drug poisoning	n(%)	71(29.3)	68(28.1)	55(22.7)	48(19.8)	242(100)
	Adjusted residuals	3.4**	−0.4	−1.0	−1.8	
Severe pneumonia	n(%)	19(13.9)	50(36.5)	36(26.3)	32(23.4)	137(100)
	Adjusted residuals	−2.4*	2.0*	0.3	−0.2	
Status epilepticus	n(%)	32(23.4)	42(30.7)	28(20.4)	35(25.5)	137(100)
	Adjusted residuals	0.6	0.4	−1.4	0.5	
Arrhythmia	n(%)	21(20.6)	21(20.6)	38(37.3)	22(21.6)	102(100)
	Adjusted residuals	−0.2	−2.0	2.9**	−0.6	
Statistic	n=970, χ²=28.877, df=12, *p*=0.004, Cramer's V=0.100					

**Note:** * Indicates the absolute value of the adjusted residual value ≥1.96 (*p* < 0.05);

** Indicates the absolute value of the adjusted residual value ≥2.58 (*p* < 0.01).

### Disease spectrum and clinical analysis of patients with CPA

During the two years of 2023–2024, 35 cases of CPA were diagnosed, accounting for 2.3% of critical illnesses. Among them, there are 14 kinds of diseases leading to CPA (see Table 6), and the top three are severe pneumonia, asphyxia, and personal injury in vehicle accidents. There were 19 males and 16 females with a median age of 4.00 (5.00, 8.00) years. The 0 to<1 year group and 6 to <12 years group accounted for 31.4% and 28.6%, respectively. The incidence of CPA outside the hospital was 77.1%, which was significantly higher than that in the hospital (25.7%). The number of patients with underlying diseases was 9 (25.7%), which was significantly lower than that of patients without underlying diseases (74.3%). According to the ROSC after cardiopulmonary resuscitation, the patients were divided into the ROSC group and the death group. There was no significant difference in gender and age between the two groups. There is no correlation between ROSC and the location of CPA, the presence or absence of underlying diseases, and the top three diseases causing CPA (Table 7).

**Table 6:** Spectrum of disorders leading to CPA.

Diseases (ICD-10 code)	n(%)
Severe pneumonia (J18.903)	5(14.3)
Asphyxia (R09.000)	4(11.4)
Multiple body region injuries (Road traffic accident) (T06.800x001)	4(11.4)
Fulminant myocarditis (I40.000x005)	3(8.6)
Shock (R57.900)	3(8.6)
Multiple injuries (Fall from height)(T07.x00)	3(8.6)
Sepsis (A41.901)	2(5.7)
Cardiac failure (I50.900)	2(5.7)
Intratracheal foreign body (T17.400)	2(5.7)
Renal insufficiency (N19.x00x002)	2(5.7)
pulmonary arterial hypertension (I27.200x012)	1(2.9)
Severe anemia (D64.903)	1(2.9)
Hypoglycemia (E16.200)	1(2.9)
Acute lymphoblastic leukemia (C91.000)	1(2.9)
Hypoxic ischemic encephalopathy (p91.600)	1(2.9)
Total	35(100)

**Table 7:** Clinical analysis of patients with CPA.

Items	Groups	Group of ROSC n(%)	Death group n(%)	Total n(%)	χ^2^	*p*
Gender	Male	8(42.1)	11(57.9)	19(54.3)	0.438	0.508
	Female	5(31.3)	11(68.7)	16(45.7)		
Age	0 to<1 yr	6(54.5)	5(45.5)	11(31.4)		0.394^*^
	1 to<3 yr	3(60.0)	2(40.0)	5(14.3)		
	3 to<6 yr	1(20.0)	4(80.0)	5(14.3)		
	6 to<12 yr	2(20.0)	8(80.0)	10(28.6)		
	12to<18 yr	1(25.0)	3(75.0)	4(11.4)		
Season	Spring	1(14.3)	6(85.7)	7(20.0)		0.497^*^
	Summer	4(40.0)	6(60.0)	10(28.6)		
	Autumn	2(33.3)	4(66.7)	6(17.1)		
	Winter	6(50.0)	6(50.0)	12(34.3)		
Site of CPA Occurrence	Out-of-hospital	11(40.7)	16(59.3)	27(77.1)		0.680^*^
	Within hospital	2(25.0)	6(75.0)	8(22.9)		
With or without underlying diseases	Without	10(38.5)	16(61.5)	26(74.3)		1.000^*^
	With	3(33.3)	6(66.7)	9(25.7)		

Note: * Indicates Fisher's exact test; yr: year/years.

## Discussion

Pediatric critical illness is characterized by abrupt onset and rapid progression, placing affected children at high risk of developing irreversible multiple organ dysfunction syndrome (MODS) or even death within a short period of time [25,26]. Therefore, pediatric emergency physicians need to be capable of rapidly identifying critical conditions and intervening promptly to improve prognosis and reduce mortality and morbidity. A thorough analysis of the disease spectrum of pediatric emergency critical illnesses provides important guidance for optimizing clinician training, rationally allocating emergency medical resources, developing targeted prevention strategies, and improving overall treatment effectiveness [27,28].

Through retrospective analysis, this study systematically described the composition, age correlation, seasonal distribution, and clinical characteristics of special critical illnesses in children. We found that: (1) Nervous system diseases accounted for the highest proportion of critically ill children in the emergency department of the hospital, followed by trauma and accidental injury, and the third was respiratory system diseases. The top five diseases were seizure, drug poisoning, severe pneumonia, status epilepticus, and arrhythmia. (2) Disease spectrum was related to the age and season of the patients. (3) The top three diseases causing CPA were severe pneumonia, asphyxia, and multiple body region injuries (road traffic accident). There was no significant difference in clinical characteristics between ROSC patients with CPA and those who died.

Globally, there are significant differences in disease composition across different regions. For example, in a tertiary medical center in the Telangana region of India, acute febrile diseases and seizures were the most common symptoms in the emergency department [29]; In Nepal, the majority of pediatric emergency patients present with injuries and infections [30]. Studies in China have shown that respiratory diseases have long ranked first in pediatric emergency departments in China [8,11]. However, a prospective cohort study in Switzerland showed that the nervous system, trauma and respiratory system were dominant in the study of high-risk populations in pediatric emergency departments [31]. This is generally consistent with our study. Neurological diseases accounted for the highest proportion of pediatric emergency critical illnesses in our hospital. Combined with seizure and status epilepticus in the top five diseases in this study, we should pay attention to the rapid assessment of nervous system emergency and process management such as airway stabilization and seizure control in clinical practice. Trauma and accidental injuries ranked second, which is similar to the 25.1% proportion of trauma reported in a Swiss study [31]. This study further suggests that road traffic accident-related multiple/combined injuries is one of the important causes of CPA, suggesting that emergency teams need to have the ability to quickly identify traumatic cardiac arrest and provide targeted intervention. Respiratory diseases ranked third. In this study, severe pneumonia was not only one of the common critical diseases, but also an important cause of CPA. The AHA/AAP2025 guidelines clearly state that cardiac arrest in children usually does not have a primary cardiac cause, but evolves from progressive respiratory failure or shock, so early identification of respiratory distress, hypoxemia, and circulatory instability in the emergency department is essential [32].

Reviewing the historical data, the top five diseases in Pediatric Intensive Care Unit (PICU) of Shengjing Hospital of China Medical University from 2005 to 2008 were, in order, acute bronchopulmonary pneumonia, poisoning, non-traumatic intracranial hemorrhage, sepsis and epilepsy; whereas from 2009 to 2012, viral encephalitis and hand, foot, and mouth disease replaced some traditional diseases to enter the top five [33]. The data of Children's Hospital Affiliated to Kunming Medical University from 2015 to 2017 showed that severe pneumonia, febrile seizure and central nervous system infection were still in the top three [34]. The results of this study showed that the top five diagnoses of critically ill children in the emergency department of our hospital from 2023–2024 were seizure, drug poisoning, severe pneumonia, status epilepticus, and arrhythmia.

It is important to note that the confounding effects of region, climate, and demographic characteristics must be carefully considered when cross-comparing this study with the previous literature. The epidemic strains of pathogens in different regions, environmental pollution levels, traffic conditions and socio-economic background may significantly affect the distribution of disease spectrum. Thus, the observed changes in disease order may not only reflect natural evolution over time but may also be strongly driven by region-specific factors. Nevertheless, the high proportions of neurological disorders (seizure, epilepsy) and poisonings in this study are generally consistent with the trends in other large children's medical centers in China in recent years [35], suggesting the increasing importance of these diseases nationwide.

The physiological development characteristics of children at different ages determine the differences in their critical illness spectrum [36]. This study found that respiratory diseases predominated in infancy (0 to <1 year), with the proportion of severe pneumonia being significantly higher than in other age groups. This is mainly attributable to the narrow airway, poor ciliary motility, and immature immune system development in infants, which predispose them to pathogen invasion and rapid progression to severe illness [37]. Severe pneumonia in children is often associated with high-risk complications, including respiratory failure and sepsis [5,38]. Therefore, a rapid identification and triage channel for infants should be established in the emergency department, focusing on monitoring respiration and blood oxygen saturation. Once high-risk features are identified, an upgraded treatment process should be started immediately, including high-flow oxygen therapy, rapid imaging evaluation and tracheal intubation preparation, so as to reduce the risk of respiratory failure and death [5,38]. The period from early childhood to preschool (1 to <6 years) represents a high-incidence age for seizures, which is related to the incomplete development of cerebral cortex, the high excitability of neurons, and the tendency of fever to induce seizures. Most seizures occurring in this period are simple febrile seizures, with an overall favorable prognosis. However, this stage is also a susceptible period for central nervous system infections [39]. Therefore, the core of emergency evaluation is to identify the few high-risk conditions that require further investigation, while avoiding unnecessary laboratory tests and Cranial Computed Tomography (CT) [40]. In school-age children (6 to <12 years), a febrile seizures and recurrent epileptic seizures are more common. Emphasis should be placed on seizure types, neurological examination, and specialist evaluations such as electroencephalography (EEG) and magnetic resonance imaging (MRI), in order to determine whether an epilepsy syndrome or other organic etiology is present [41]. Among adolescent patients (12 to <18 years), drug poisoning accounted for the highest proportion, which was consistent with the findings of Borensztajn et al. [42] and Wang Xiao et al. [43]. It should be emphasized that this study only recorded the clinical diagnosis of “drug poisoning” and did not collect data on patients’ intention to take medication (intentional self-harm vs accidental ingestion), psychological assessment, or social background. Although previous literature often mentions that academic pressure, rebellious psychology and other factors are related to drug abuse in adolescents [44,45]. However, in the absence of specific data on intention, the high incidence of drug poisoning cannot be directly equated with an “adolescent psychological crisis.” The observed high rate is more likely to reflect increased accessibility of medications to adolescents and their weak risk awareness. Therefore, the current clinical focus should be placed on improving early identification of the risk of drug overdose in adolescents, standardizing detoxification treatment, and strengthening the management of medication safety at home.

Seasonal variation has a significant impact on the occurrence of pediatric critical illnesses. In this study, cases of drug poisoning were more concentrated in spring, while the study by Hu Juan et al. [46] suggested that accidental ingestion incidents were more common in summer. This difference may be related to regional climate, lifestyle habits, and the intensity of supervision. The seasonal distribution of severe pneumonia also exhibits dynamic changes. Li Cuilian et al. [34] reported that it was more common in winter and spring, whereas our data showed that severe pneumonia in children was more prevalent in summer in 2023 and 2024. This may be related to the prevalence or outbreak of specific pathogens, with certain viruses and bacteria being more common in summer, such as *adenovirus, parainfluenza virus*, *Streptococcus pneumoniae* [47]. In addition, in northern China, the large indoor-outdoor temperature difference caused by air conditioning use and crowd gathering in summer may also promote the transmission of respiratory diseases. However, other possibilities should be acknowledged, such as differences in the diagnostic criteria for “severe pneumonia” among clinicians and variations in the definition of “severe pneumonia” across different years, which may lead to fluctuations in the constituent ratio [10]. Furthermore, this study only analyzed data from 2023 and 2024, which may introduce data bias. The seasonal distribution of seizures and status epilepticus was not significant, which was consistent with previous studies [34]. Understanding the seasonal distribution of pediatric critical illnesses has important practical implications for emergency department resource allocation, staff scheduling, bed planning, and optimization of critical care processes.

CPA is a serious outcome in critical diseases. In this study, the top three diseases leading to CPA were severe pneumonia, asphyxia, and multiple body region injuries (road traffic accident). There was no significant difference in clinical characteristics between ROSC patients with PCA and those who died. Shepard LN et al. showed that the diseases with PCA were mainly respiratory diseases and heart diseases. Patients who achieved ROSC were older, and there was no difference in gender, race, or ethnicity between ROSC patients and death patients [21]. This finding suggests that regardless of the initial etiology, once progression to CPA, the prognosis is poor, and emphasizes the importance of early identification of high-risk signs and prevention of CPA.

The main strengths of this study are the detailed description of the disease spectrum characteristics of pediatric emergency critical illness cases, and the in-depth analysis combined with age and season dimensions, which provides an empirical basis for the allocation of localized emergency resources. However, this study has several limitations. First, as a single-center retrospective study, it only included data from 2023 to 2024, which may not be sufficient to reflect long-term epidemiological trends. Second, the data relied on electronic medical records, where diagnoses were recorded by individual physicians without a standardized protocol, and the heterogeneity in diagnostic coding may introduce misclassification bias. In addition, this study used the International Classification of Diseases, 10th Revision (ICD-10) for disease classification. Given that the World Health Organization has released ICD-11, which provides a more refined classification structure and better data interoperability, continued use of ICD-10 may fail to capture nuances in certain disease subtypes and may result in mapping bias when compared with other databases adopting the new standard. Finally, our study did not include outcome or follow-up data, and therefore could not evaluate long-term prognosis or intervention effects.

## Conclusion

This study suggests that nervous system, trauma and accidental injury, and respiratory system are dominant in children's emergency critical illness, and there are obvious age and season clustering characteristics. It is suggested that customized management strategies based on age and season be adopted for common critical illnesses, in order to improve the efficiency of treatment and the targeted utilization of resources. At the same time, combined with the characteristics that the causes of CPA in this study are concentrated in severe pneumonia, asphyxia and multiple body region injuries (road traffic accident), emergency department must maintain standardized, universally applicable emergency response protocols to ensure rapid initiation of high-quality CPR in all scenarios. The findings also highlight the need to strengthen targeted training for emergency staff (including season- and age-specific skills) and optimize resource allocation (e.g., dynamic deployment of equipment, medications, and personnel). We recommend that hospital administrators and emergency department heads take the lead in implementing these measures, with support from local health authorities. These results provide evidence for refining critical care management strategies in the emergency department, and future multi-center studies are needed to validate the impact of customized approaches on clinical outcomes.
